# Preparation of CL-20 with Controllable Particle Size Using Microfluidic Technology

**DOI:** 10.3390/molecules30051176

**Published:** 2025-03-06

**Authors:** Zihao Zhang, Jin Yu, Yujia Wen, Hanyu Jiang, Siyu Xu, Yubao Shao, Ergang Yao, Heng Li, Fengqi Zhao

**Affiliations:** Xi’an Modern Chemistry Research Institute, Xi’an 710065, China; zzh565399203@stu.xjtu.edu.cn (Z.Z.); yujinnayo@163.com (J.Y.); 18391853059@163.com (Y.W.); jianghanyu612@126.com (H.J.); 18040119707@163.com (Y.S.); yaoerg@126.com (E.Y.); zerolhheart@163.com (H.L.); npecc@163.com (F.Z.)

**Keywords:** CL-20, microfluidic, multiple particle size, recrystallization

## Abstract

As a typical high-energy-density material, the sensitivity of CL-20 severely limits its application in explosives and propellants. Adjusting its structure at the microscopic level can effectively solve such problems. In this study, a microfluidic recrystallization technique was used to prepare ε-CL-20 with three different particle sizes, with narrow particle size distributions (D_50_ = 2.77 μm, 17.22 μm and 50.35 μm). The prepared samples had fewer surface defects compared to the raw material. As the particle size decreased, the density of CL-20 increased and its impact sensitivity was significantly reduced. The activation energy of the CL-20 prepared using microfluidic technology increased with increases in particle size. Laser ignition experiments revealed that smaller CL-20 particles had the highest energy release efficiency, while larger particles exhibited a higher energy density and more stable energy release. The combustion performance and safety of CL-20 can be effectively improved by improving the crystal size distribution and surface morphology. Controllable preparation of multiple particle sizes of CL-20 was achieved using microfluidic recrystallization technology, which provides a reference for the preparation of multiple particle sizes of other energetic materials.

## 1. Introduction

2,4,6,8,10,12-hexanitro-2,4,6,8,10,12-hexaazaisowurtzitane (CL-20 or HNIW) is a cage nitramine compound [1,2]. CL-20 has a high density (2.04 g·cm^−3^) and detonation speed (9400 m·s^−1^), making it the preferred material for propellants and composite explosives [3,4]. However, the high sensitivity of CL-20 and its easy crystallization during the preparation process seriously limit its practical application [5,6]. Reducing the sensitivity of CL-20 without reducing its energy properties has become a focus of research. Studies have shown that the morphology, particle size, and particle size distribution are the main factors affecting the performance of CL-20 [7]. Wang et al. [8] prepared submicron β-CL-20 particles using ultrasonic spray-assisted electrostatic adsorption technology. Compared with the raw materials, the thermal decomposition efficiency of submicron CL-20 particles had been improved. Zhang Pu and Li et al. [9,10] prepared spherical ultrafine CL-20 using a grinding method, and found that the mechanical sensitivity of the ground CL-20 was better than that of the raw material. Liu and Kalman et al. [11,12] studied the effect of CL-20 particle size on propellant combustion performance. It is found that with an increase in particle size, the thermal stability of CL-20 became better and the ignition delay time increased. The above conclusions indicate that the application performance of CL-20 can be effectively improved by adjusting the particle size and improving the particle size distribution of CL-20.

At present, the development of microfluidic technology provides a new method for the preparation of energetic materials. The combination of microfluidic technology and solvent–non-solvent recrystallization makes the crystallization process more controllable. The microfluidic recrystallization process occurs in microchannels, and the homogeneity of solution saturation is good. Moreover, it has the advantages of high heat and mass transfer efficiencies, high safety, and accurate parameter regulation [13,14,15,16,17,18,19]. Therefore, microfluidic technology provides a reliable method for the preparation of CL-20 with various particle sizes and a narrow particle size distribution, and contributes to the high-quality preparation of CL-20.

In this study, CL-20 was prepared using microfluidic recrystallization. The uniform supersaturation was leveraged to achieve a narrow size distribution for the CL-20 particles. The morphology and size of the CL-20 particles were controlled by adjusting the process parameters. The characteristics of the particles, including their morphology, size, and polymorph transformation, were analyzed using SEM and XRD. Subsequently, the thermal decomposition, density, impact sensitivity, and electrostatic spark sensitivity of the samples were evaluated. The pyrolysis and safety performance of the CL-20 particles of different sizes were investigated and analyzed. Finally, the combustion behavior of the CL-20 with multiple particle sizes was examined through laser ignition experiments.

## 2. Results and Discussion

### 2.1. Morphology Analysis

**Figure 1:**
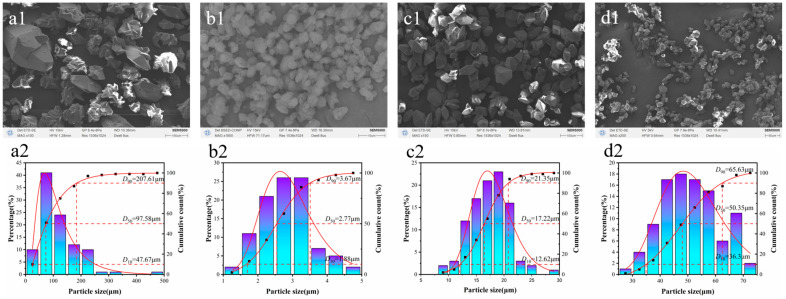
SEM and statistical plots of particle size. (**a**) Raw CL-20, (**b**) S1, (**c**) S2, (**d**) S3. (**a1**,**a2**) SEM and particle size distribution of Raw CL-20; (**b1**,**b2**) SEM and particle size distribution of S1; (**c1**,**c2**) SEM and particle size distribution of S2; (**d1**,**d2**) SEM and particle size distribution of S3.

CL-20 particles with different sizes were prepared using microfluidic recrystallization, and their morphology and particle size distribution were characterized. The scanning electron microscopy (SEM) images and particle size distributions of the raw CL-20 and the prepared samples (S1, S2, and S3) are presented in Figure 1.

As shown in Figure 1, the particle size distributions of the three samples become narrower after microfluidic recrystallization. The D_50_ of S1, S2, and S3 changed from 97.58 μm (raw material) to 2.77 μm, 17.22 μm, and 50.35 μm, respectively. The Span value of the raw material particle size distribution was 1.64, and the Span values of the S1, S2, and S3 particle size distributions were 0.65, 0.51, and 0.58, respectively. The particle size distribution of CL-20 was obviously improved after recrystallization. During the microfluidic recrystallization process, the uniformity of the solution supersaturation within the microchannel ensures uniform nucleation and crystal growth. The absence of significant supersaturation gradients in the microchannel effectively prevents explosive nucleation, leading to a well-defined crystal morphology and narrow particle size distribution.

**Figure 2:**
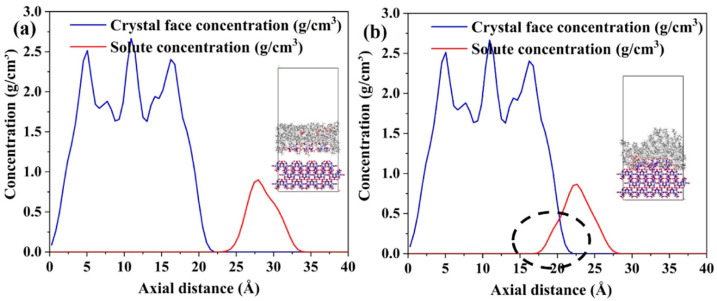
Axial concentration distribution of solute on the (0 0 1) crystal face (**a**) before adsorption; (**b**) after adsorption [21]. (The black dashed box indicates that the axial concentration distribution curve of the ε-CL-20 molecule intersects the concentration curve of the crystal surface, indicating that some solutes have entered the groove area of the crystal face).

Compared with the raw material, the particle size of the CL-20 crystals decreased significantly after microfluidic recrystallization. This was due to the high concentration of CL-20 in the solution (0.3 g·ml^−1^), large flow ratio of the solvent to anti-solvent (1:10~1:20), high supersaturation, and good uniformity. According to the Gibbs–Thomson equation [20], there is an inverse relationship between the critical radius of the crystal nucleus and supersaturation. Therefore, small spheroidal β-CL-20 crystals were obtained at the collection unit, and after polymorphic transformation, ε-CL-20 crystals with a narrow particle size distribution were produced. Moreover, when n-octane was used as the anti-solvent, ε-CL-20 crystals with a D_50_ of 2.77 μm were obtained after treatment at 323.15 K for 15 min. The analysis showed that this was due to the large difference in boiling points between the solvent and anti-solvent. The boiling points of the three reagents used in the experiment are shown in Table 1, which shows that the boiling point of n-octane is significantly higher than that of ethyl acetate and petroleum ether. After mixing the solvent and anti-solvent in the microchannel, β-CL-20 crystals with a particle size of 0.5~1 μm were immediately collected from the suspension in the beaker. In the ethyl acetate–n-octane solvent system, the large difference in boiling points caused ethyl acetate to evaporate rapidly at 323.15 K, while the volume of n-octane remained largely unchanged. This process drove the supersaturation of CL-20 in the suspension to close to 100%, thereby halting crystal growth at a smaller particle size. As shown in Figure 2, a high supersaturation effectively accelerated the polymorphic transformation of CL-20, facilitating the formation of smaller ε-CL-20 crystals [21]. The results demonstrate that microfluidic technology can effectively produce ε-CL-20 crystals with multiple particle sizes.

In the ethyl acetate–petroleum ether system, stirring the suspension accelerated the dissolution of the metastable β-form crystals, promoting the growth of the stable ε-form and reducing the time required for polymorphic transformation. The subsequent settling allowed for uniform crystal growth, resulting in well-formed ε-CL-20 crystals. The particle size of the crystals also increased with increasing standing time. It was found that during the settling process, the slow evaporation of ethyl acetate and petroleum ether led to more CL-20 precipitating out. The initially formed ε-CL-20 crystals during the stirring acted as seed crystals, inducing further growth of CL-20 on their surfaces and ultimately yielding larger crystals [22].

### 2.2. XRD Analysis

**Figure 3:**
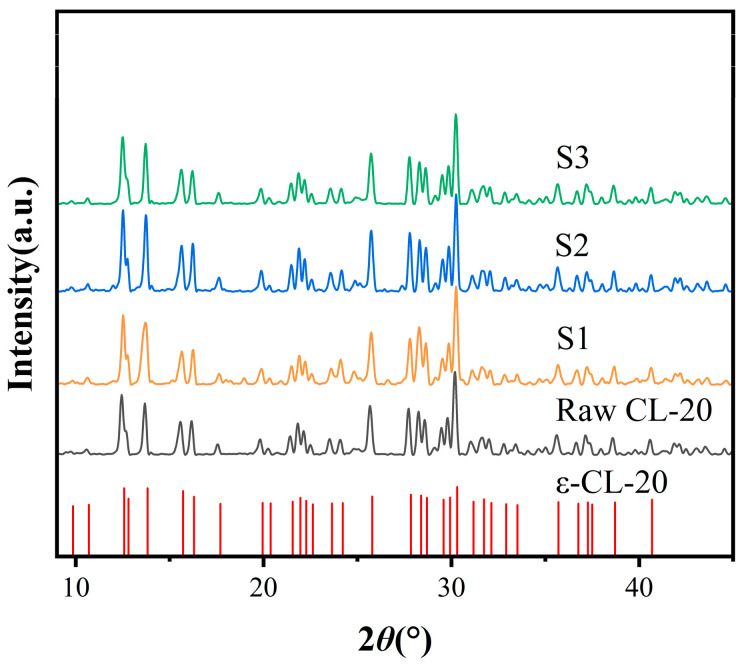
XRD of raw CL-20, S1, S2, and S3.

To determine the crystal structure, the raw materials and three samples were analyzed by XRD. The results are shown in Figure 3. The characteristic diffraction peaks of the raw material and the three samples all appeared at 10.7°, 12.6°, 15.75°, 16.35°, 25.85°, and 30.4°, corresponding to the standard diffraction peaks shown in PDF#50-2045, indicating that the raw material and the three samples were ε-CL-20 (characteristic diffraction peaks of α-CL-20: 12.124°, 13.761°, 13.948°, 15.075°, 17.558°, and 20.199°, corresponding to the standard diffraction peaks shown in PDF#52-2431; characteristic diffraction peaks of β-CL-20: 7.613°, 13.718°, 24.187°, 24.904°, 28.306°, and 30.309°, corresponding to the standard diffraction peaks shown in PDF#52-2432).

The initial recrystallization process of CL-20 was governed by solvent kinetics, and the nucleation energy of β-CL-20 was generally lower than that of ε-CL-20. According to Ostwald’s rule, the growth rate of metastable β-CL-20 was much higher than that of thermodynamically stable ε-CL-20, leading to the initial precipitation of metastable β-CL-20 in the solution. During the recrystallization process, solution-mediated polymorphic transformation occurred. As steady-state ε-type crystal nuclei began to form and grow, the concentration of the solute in the solution decreased. This caused the metastable β-form to dissolve while the stable form continued to grow, ultimately resulting in the most thermodynamically stable ε-form [23,24]. In the sample preparation process, heating or stirring of the suspension accelerated the dissolution of metastable β-form crystals, promoted the growth of the stable ε-form, and shortened the time required for polymorphic transformation. The subsequent settling allowed for uniform crystal growth, leading to the formation of well-formed ε-CL-20 crystals. Additionally, the absence of solvents with high dipole moments in the experiment ensured that the crystal form of CL-20 did not transform into the α-CL-20 form. Moreover, since the entire experimental process was conducted at room temperature, the crystal form of CL-20 remained stable and did not convert to the γ-CL-20 form.

### 2.3. Dispersion Performance and True Density Analysis

**Figure 4:**
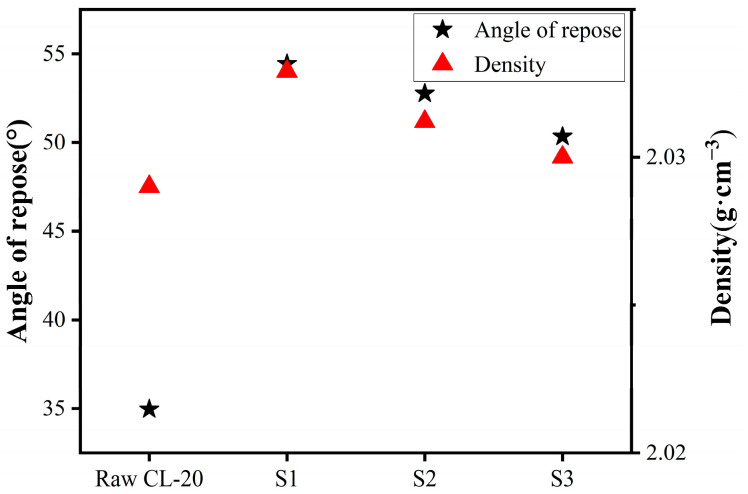
The angle of repose and density of raw CL-20, S1, S2 and S3.

The angle of repose method was used to test the dispersion of the raw material and three samples (S1, S2, and S3). The experimental results are shown in Figure 4. The angle of repose of the raw material was 34.96°, and the angles of repose of the three samples were 54.43°, 52.76°, and 50.33°, respectively. Compared with the raw material, their dispersibility decreased. It was found that with an increase in particle size, the dispersion of particles gradually increased. This was because when the particles are small, the surface energy is larger, which leads to the agglomeration of particles, making the dispersion performance worse.

The true density of the samples was measured using the gas displacement method, and each sample was measured seven times and the average value was analyzed. The true density of the raw material was 2.029 g·cm^−3^, which was the lowest compared with the other three samples. The true density was mainly affected by crystal growth and defects. Compared with the three samples, the raw CL-20 had more surface defects, and the uneven growth may have led to more internal defects. These factors resulted in the low true density of raw CL-20. The packing densities of S1, S2, and S3 were 2.0329 g·cm^−3^, 2.0312 g·cm^−3^ and 2.0302 g·cm^−3^, respectively, which were higher than that of raw CL-20 (Figure 4). It was found that the S1, S2, and S3 sample crystals had fewer surface defects. During the growth of the crystals, due to the different growth rates of each crystal face of CL-20, there will be some gaps in the crystal, causing the true density to decreases with increasing particle sizes.

### 2.4. Thermal Performance Analysis

**Figure 5:**
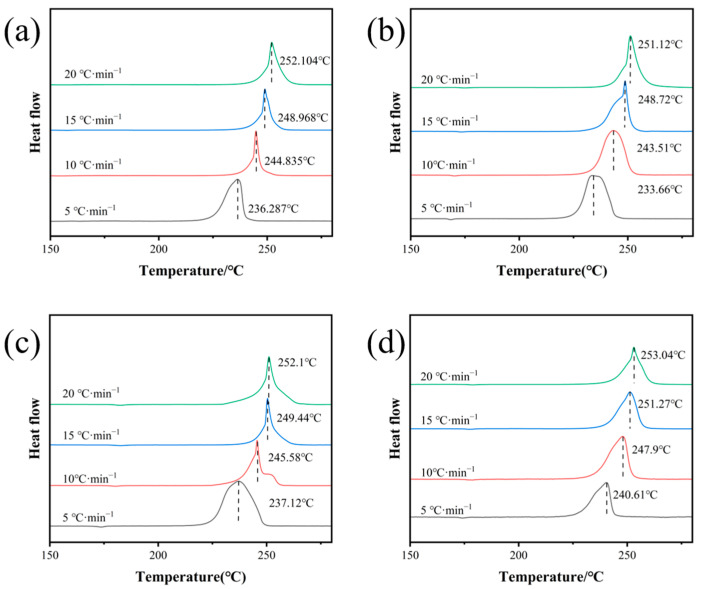
DSC curves of raw CL-20, S1, S2, and S3. (**a**) Raw CL-20, (**b**) S1, (**c**) S2, (**d**) S3.

To investigate the thermal decomposition performance and thermal safety of CL-20, S1, S2, and S3, differential scanning calorimetry (DSC) was used to analyze the raw CL-20 and three samples (S1, S2, and S3). The results are shown in Figure 5. As the particle size increased, the thermal decomposition peak temperatures of S1, S2, and S3 also increased. This trend was attributed to the larger specific surface area of smaller particles, which facilitates faster heat transfer between particles and consequently lowers the thermal decomposition peak temperature. Compared to raw CL-20, the decomposition peak temperature of S1 decreased by 1.32 K, while those of S2 and S3 increased by 0.75 K and 3.06 K, respectively. The analysis suggested that the microfluidic recrystallization process improved the morphology of the samples, eliminating large surface defects. This enhancement effectively reduced the accumulation of hot spots, thereby improving the thermal safety performance.

(1) $${Tp}_{i}={Tp}_{0}+b\beta_{i}+c\beta_{i}^{2}+d\beta_{i}^{3}$$

(2) $$T_{b}=\frac{E_{a}-\sqrt{{E_{a}}^{2}-\;4RE_{a}Tp_{0}}}{2R}$$

In this equation, *T_pi_* represents the decomposition temperature at different heating rates (K); *T_b_* represents the thermal explosion critical temperature (K); and *E_a_* is the apparent activation energy calculated using the Kissinger and Ozawa equation (kJ·mol^−1^).

The thermodynamic and kinetic parameters of energetic materials are important indicators of their thermal decomposition characteristics. The thermal decomposition kinetic parameters of the raw material and the three samples were calculated using the Ozawa method and the Kissinger method, with the results shown in Table 2. As the particle size increased, the apparent activation energy of the particles also increased. The activation energy of all the samples except S1 was higher than that of the raw material, indicating that the particles with more regular surface morphology needed more energy to transform from a steady state to an active state. Additionally, the *Tp*_0_ (peak temperature when the heating rate approaches zero) and *T_b_* (thermal explosion critical temperature) of the three samples were equal to or higher than those of the raw CL-20. These results indicate that CL-20 with the three particle sizes exhibited excellent thermal stability [25,26].

### 2.5. Analysis of Combustion Behavior

**Figure 6:**
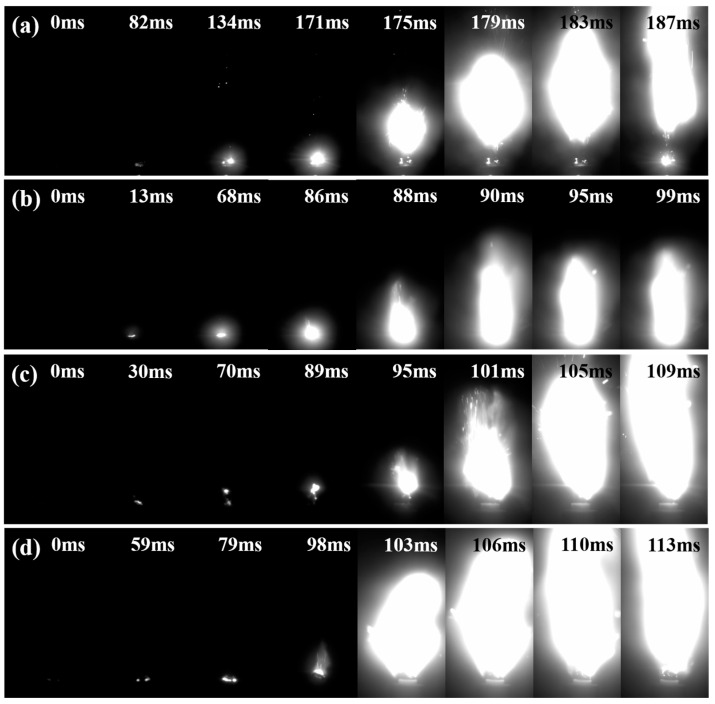
Images of flame propagation at 192 W/cm^2^ for (**a**) raw CL-20, (**b**) S1, (**c**) S2, (**d**) S3.

**Figure 7:**
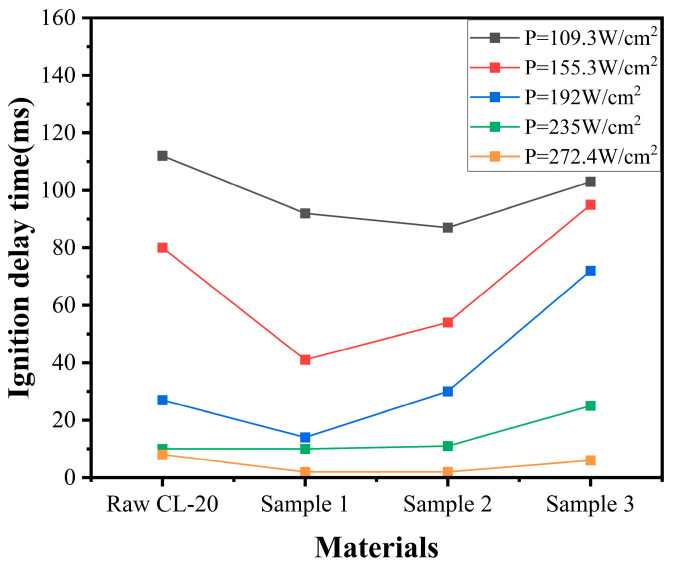
Ignition delay time of raw CL-20, S1, S2, and S3.

To evaluate the combustion performance of raw CL-20 and the three samples, they were mixed with adhesive GAP in a specific ratio and subjected to laser ignition tests. High-speed cameras were used to record the combustion flames and ignition delay times, with the results presented in Figure 6 and Figure 7. After microfluidic recrystallization, it was observed that smaller particle sizes resulted in shorter ignition delay times and the faster formation of noticeable flames. This was attributed to the larger specific surface area of the prepared CL-20 particles, which enhanced the heat transfer efficiency between particles. Additionally, smaller particles possess lower activation energies, allowing them to more readily reach an excited state. These factors collectively contributed to the reduced ignition delay times and faster flame formation. In contrast, the large CL-20 particles prepared by microfluidic recrystallization had a higher activation energy. As a result, more energy is required to initiate the reaction, leading to a slower combustion rate and a more diffuse flame structure [27]. Consequently, larger particles produced larger flames and exhibited faster flame propagation perpendicular to the combustion surface. Since the activation energies of S2 and S3 were higher than that of the raw CL-20, their flames were significantly larger than those of the raw CL-20.

### 2.6. Sensitivity Analysis

**Figure 8:**
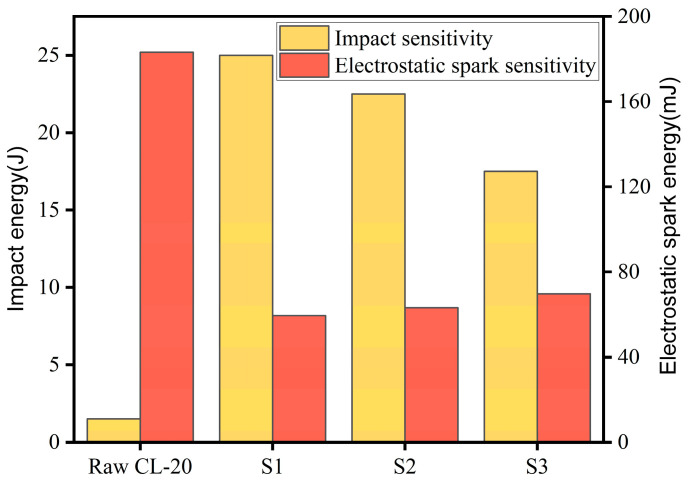
Impact sensitivity and electrostatic spark sensitivity of raw CL-20, S1, S2, and S3.

The impact sensitivity and electrostatic spark sensitivity of raw CL-20, S1, S2, and S3 were tested, and the results are shown in Figure 8. The impact energy values of raw CL-20, S1, S2, and S3 were 1.5 J, 25 J, 22.5 J, and 17.5 J, respectively. Compared to the raw material, the impact energy of the three recrystallized samples was significantly improved, with smaller particles exhibiting higher impact energies. This improvement was attributed to the better surface morphology and fewer crystal defects in the microfluidically prepared particles. These characteristics effectively reduce hot spots caused by impacts and friction between particles. For the same mass, smaller particles have a larger number of individual particles, which increases the heat transfer efficiency between them. As a result, the energy of the generated hot spot can be released in time, making it difficult for the hot spot to grow [28].

The electrostatic spark energy values of raw CL-20, S1, S2, and S3 were 183.25 mJ, 59.51 mJ, 63.19 mJ, and 69.68 mJ, respectively. The electrostatic spark sensitivity of CL-20 decreased with an increase in particle size. The specific surface area of the small particles prepared using microfluidic technology was larger, so more charge accumulated on the surface of the particle. On the other hand, the apparent activation energy of the small particles was also low, so it was easy to reach the excited state under lower energy stimulation, making its electrostatic spark energy lower.

## 3. Materials and Methods

### 3.1. Experimental Materials and Facilities

**Figure 9:**
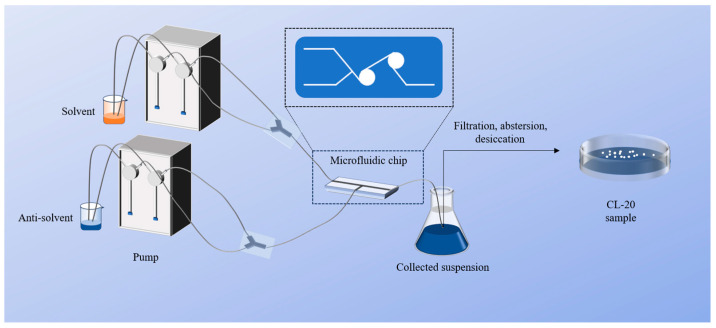
The microfluidic system.

The following reagents and equipment were used: raw CL-20 (Xi’an Modern Chemistry Research Institution, Xi’an, China); ethyl acetate (AR grade; Sinopharm Chemical Reagent Co., Ltd., Shanghai, China); petroleum ether (AR grade; Sinopharm Chemical Reagent Co., Ltd.); n-octane (AR grade; Sinopharm Chemical Reagent Co., Ltd.); a peristaltic pump (Sanotac Co., Ltd., Münster, Germany); a T-type microfluidic chip (Microflu Co., Ltd., Changzhou, China); a magnetic stirrer (IKA Co., Ltd., Wilmington, NC, USA); and a water bath (Senco Co., Ltd., Cincinnati, OH, USA). The microfluidic system was composed of a drive module, a recrystallization module, and a collection module, as shown in Figure 9.

### 3.2. Preparation of CL-20 with Multiple Particle Sizes

The preparation of CL-20 with various particle sizes is shown in Figure 1. The microfluidic preparation system used consisted of a driving module, a recrystallization module, and a collection module. The injection pump is used as the driving device for conveying liquid, the vortex microchannel is used for producing particles, and the beaker is used for collecting the suspension. Ethyl acetate was selected as the solvent to prepare the CL-20 solution with a concentration of 0.3 g·ml^−1^, which was then heated to 323.15 K in a water bath. Petroleum ether and n-octane were selected the anti-solvent. CL-20 particles with different particle sizes were prepared by adjusting the flow ratio, crystallization temperature, treatment method, and settling time of the suspension. When the drive module started working, the solvent and the anti-solvent were rapidly mixed in the recrystallization module to produce white particles, and then the suspension was collected for further processing. After the suspension was treated for a period of time, it was washed 3–5 times with anhydrous ethanol, filtered, and dried to obtain S1, S2, and S3 (Table 3). This preparation method is simple to perform and can precisely control the crystallization process according to the required specification, which has significant advantages in the safe preparation of energetic materials.

### 3.3. Characterization

The morphology of CL-20 was observed using scanning electron microscopy (SEM, FEI JSM-5800) (CIQTEK Co., Ltd., Hefei, China), and the particle size distribution of the sample was analyzed using Nanomesure software (v1.2.5). The structure of raw CL-20 and the samples were confirmed by X-ray powder diffraction (XRD; PANalytical Empyrean diffractometer equipped with Cu-Kα emission sources operating at 40 kV and 40 mA) (Malvern Panalytical (Shanghai) Co., Ltd., Shanghai, China). Data were collected in the range of 10° to 40° (2θ), with a step size of 0.02°. The standard spectra of α-, β-, γ-, and ε-CL-20 at ambient temperature were obtained from the ICDD database.

Thermal Property Analysis: The non-isothermal decomposition experiments on the raw material and three samples were conducted using a Differential Scanning Calorimeter (DSC, Setline) (KEP Technologies (Shanghai) Co., Ltd., Shanghai, China) In each experiment, 0.3–0.5 mg of the sample was weighed on a precision balance (0.01 mg, Sartorius SQP, Göttingen, Germany) and placed into an aluminum (Al) crucible. The temperature range was set from 25 °C to 300 °C, with heating rates of 5, 10, 15, and 20 °C·min^−1^.

Impact sensitivity test: The characteristic height of raw CL-20 and the three samples was calculated using the “characteristic height method” and an impact sensitivity meter. The test conditions were as follows: each sample was tested 25 times, a single dose was 40 mg, the drop weight was 5.0 kg, the ambient temperature was 10 °C, and the relative humidity was 60%.

Electrostatic spark sensitivity test: The electrostatic spark sensitivities of the raw CL-20 and samples were tested using the HT-20IB-3 electrostatic spark sensitivity meter developed by Hubei Institute of Aerospace Chemistry and Technology according to the aerospace industry standard QJ 20019.5-2018 (“Test Method for Safety Performance of Composite Solid Propellant: Electrostatic Spark Sensitivity”) [29].

## 4. Conclusions

In this research, ε-CL-20 with three particle sizes was successfully prepared by microfluidic recrystallization. The D_50_ values were 2.77 μm, 17.22 μm, and 50.35 μm, respectively. The Span values of the particle size distribution were 0.65, 0.51 and 0.58, respectively, indicating narrow and well-controlled size distributions for all the samples. The main research results for the raw material and the three samples included those from angle of repose tests, density tests, thermal decomposition tests, ignition delay time tests, and sensitivity tests. The results revealed that the microstructure of the CL-20 samples primarily exhibited a spindle shape, with smaller particles tending toward a more spherical morphology. As the particle size increased, the dispersion of CL-20 improved. Smaller particles exhibited higher densities, while larger crystals demonstrated superior thermal stability, making thermal decomposition more difficult to initiate. The ignition delay time and flame intensity also increased with particle size. Compared to the raw material, the prepared CL-20 samples exhibited significantly reduced impact sensitivities. The impact sensitivity increased with particle size, but the electrostatic spark sensitivity decreased. This study demonstrates that it is feasible to improve the combustion performance and safety of CL-20 by altering its microstructure through microfluidic technology. It provides a simple, efficient, and safe strategy for preparing ε-CL-20 with controlled particle sizes, offering valuable insights for the development of high-performance energetic materials.

## Figures and Tables

**Table 1:** Boiling points of the reagents.

Reagent	Ethyl Acetate	Petroleum Ether	Normal Octane
Boiling point/K	349.65	333.15	399.55

**Table 2:** Thermal kinetic parameters of raw CL-20 and samples.

Sample	Kissinger Method		Ozawa Method		*Tp_0_*/°C	*T_b_*/°C
	*E_a_*	R^2^	*E_a_*	R^2^		
Raw CL-20	176.38	0.9913	185.06	0.9973	219.91	222.12
S1	162.2	0.9933	152.33	0.9891	217.34	219.81
S2	194.14	0.9939	185.93	0.9926	220.66	222.74
S3	226.02	0.9869	232.26	0.9888	227.08	228.96

**Table 3:** Process parameters for the preparation of CL-20 with various particle sizes.

Sample	Solvent	Concentration/g·mL^−1^	Anti-Solvent	Flow Ratio	Post-Processing Method and Time
S1	Ethyl acetate	0.3	Normal octane	1:20	Treatment at 50 °C for 15 min
S2	Ethyl acetate	0.3	Petroleum ether	1:15	Stir for 1 h and stand for 4 h
S3	Ethyl acetate	0.3	Petroleum ether	1:10	Stir for 1 h and stand for 6 h

S1—Sample 1; S2—Sample 2; S3—Sample 3.

## Data Availability

The data presented in this study are available on request from the corresponding author.
